# Drug-Resistant Tuberculosis, Georgia, Kazakhstan, Kyrgyzstan, Moldova, and Ukraine, 2017–2022 

**DOI:** 10.3201/eid3004.231732

**Published:** 2024-04

**Authors:** Victor Naestholt Dahl, Tetiana Butova, Alex Rosenthal, Alina Grinev, Andrei Gabrielian, Sergo Vashakidze, Natalia Shubladze, Bekzat Toxanbayeva, Lyailya Chingissova, Valeriu Crudu, Dumitru Chesov, Gulmira Kalmambetova, Gulbarchyn Saparova, Christian Morberg Wejse, Dmytro Butov, Ukraine TB-Portal Study Group

**Affiliations:** Aarhus University Hospital, Aarhus, Denmark (V.N. Dahl, C.M. Wejse);; Merefa Central District Hospital, Merefra, Ukraine (T. Butova);; National Institutes of Health, Bethesda, Maryland, USA (A. Rosenthal, A. Grinev, A. Gabrielian);; National Center for Tuberculosis and Lung Diseases, Tbilisi, Georgia (S. Vashakidze, N. Shubladze);; National Scientific Center of Phthisiopulmonology, Almaty, Kazakhstan (B. Toxanbayeva, L. Chingissova);; Institute of Phthisiopneumology, Chisinau, Moldova (V. Crudu);; State University of Medicine and Pharmacy, Chisinua (D. Chesov);; National TB Program, Bishkek, Kyrgyzstan (G. Kalmambetova, G. Saparova);; Kharkiv National Medical University, Kharkiv, Ukraine (D. Butov)

**Keywords:** tuberculosis and other mycobacteria, bacteria, multidrug-resistant tuberculosis, extensively drug-resistant tuberculosis, treatment outcome, disease management, epidemiology, Eastern Europe, Central Asia, Georgia, Moldova, Ukraine, Kazakhstan, Kyrgyzstan

## Abstract

In 2021, the World Health Organization recommended new extensively drug-resistant (XDR) and pre-XDR tuberculosis (TB) definitions. In a recent cohort of TB patients in Eastern Europe, we show that XDR TB as currently defined is associated with exceptionally poor treatment outcomes, considerably worse than for the former definition (31% vs. 54% treatment success).

In early 2021, the World Health Organization (WHO) recommended new definitions of extensively drug resistant (XDR) and pre-XDR tuberculosis (TB) (*1*,*2*). Previously, pre-XDR TB was informally defined as TB caused by *Mycobacterium tuberculosis* strains with resistance to rifampin and isoniazid plus resistance to either a fluoroquinolone or a second-line injectable, but not both (*1*–*3*). Now, pre-XDR TB is officially defined as strains with resistance to rifampin, isoniazid, and a fluoroquinolone (levofloxacin or moxifloxacin), whereas XDR TB is now defined as additional resistance to >1 group A drug (bedaquiline or linezolid), replacing the second-line injectables used in the former definitions (*1*–*3*).

Treatment outcomes of patients with XDR TB as currently defined have been sparsely reported. A study from France with 93 patients fulfilling the new pre-XDR TB and XDR TB definitions, including 9 patients with XDR TB, found a combined treatment success of 68% (n = 63), comparable to that of multidrug-resistant (MDR) TB (*4*). Another study following 9 XDR TB patients from Georgia documented a treatment success of only 22% (n = 2/9) (*5*). We describe MDR TB, pre-XDR TB, and XDR TB treatment outcomes in Georgia, Kazakhstan, Kyrgyzstan, Moldova, and Ukraine during 2017–2022 in patients with drug susceptibility tests available for fluroquinolones, second-line injectables, bedaquiline, and linezolid.

Using prospectively collected data from the National Institute of Allergy and Infectious Diseases TB Portals Program, as described elsewhere (*6*), we included 1,960 patients with MDR TB, pre-XDR TB, and XDR TB in the analysis. Median age was 43 (interquartile range 35–51) years; 78% (1,535) were men and 22% (425) women; 63% (1,235) were smokers, and 18% 350) were persons with HIV. Most patients were from Ukraine (n = 1,455), Moldova (n = 289), and Georgia (n = 160), whereas only a few were from Kazakhstan (n = 39) and Kyrgyzstan (n = 17).

Of the 1,960 patients, 36% (698) were classified in a different category using the current definitions than for the previous definitions; XDR TB accounted for a much smaller percentage (2.7%, 95% CI 2.0%–3.5%) of patients than under the previous definition (18.5%, 95% CI 16.8%–20.3%). Using WHO treatment outcomes (*6*,*7*), our results showed that the current XDR TB definition was associated with low treatment success (sum of treatment completed and cured), only 31% (95% CI 19%–45%), compared with 54% (95% CI 49%–59%) using the former definition (p = 0.002 by χ^2^ test) (Figure). That finding was mainly driven by a higher percentage of failure using the current definition (33%, 95% CI 21%–47%) than when using the former (15%, 95% CI 11%–19%; p = 0.001) and a lower percentage of cured (25% [95% CI 14%–39%] vs. 46% [95% CI 41%–51%]; p = 0.004). Although a history of TB among patients with XDR TB was associated with a notably lower percentage of successful outcomes (23%, 95% CI 11%–41%) compared with the percentage of successful outcomes in persons without a history of TB (47%, 95% CI 24%–71%), this difference did not reach statistical significance (p = 0.076). MDR TB and pre-XDR TB treatment outcomes were comparable for both definitions.

**Figure:**
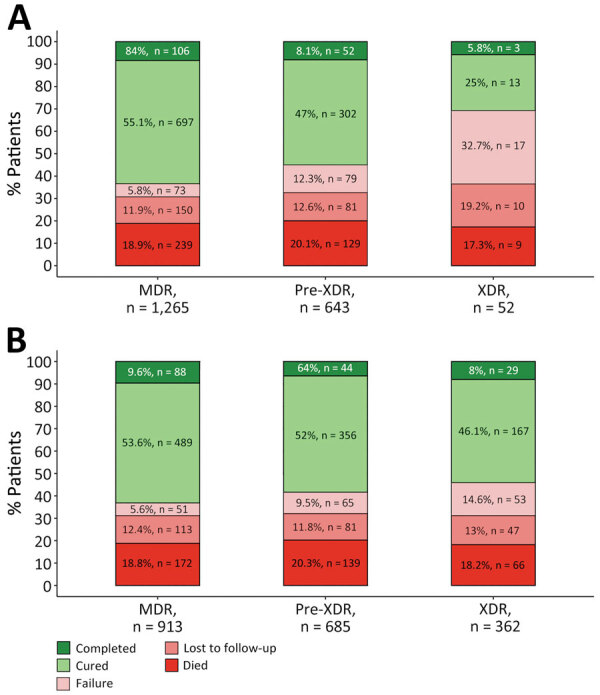
Treatment outcomes for patients with MDR, pre-XDR, and XDR tuberculosis (TB) in Georgia, Kazakhstan, Kyrgyzstan, Moldova, and Ukraine during 2017–2022 by current (A) and former (B) definitions of drug resistance. We excluded 9 patients with an unevaluated outcome and 15 patients without outcome data. TB treatment outcomes were defined according to WHO recommendations (*6*,*7*). MDR TB was defined as TB caused by *Mycobacterium tuberculosis* strains resistant to at least both rifampin and isoniazid (*1*). We used the current definition of pre-XDR TB from 2021 as TB caused by *M. tuberculosis* strains fulfilling the definition of MDR TB but including resistance to any fluoroquinolone (levofloxacin or moxifloxacin), whereas XDR TB was defined as additional resistance to >1 group A drug (bedaquiline or linezolid) (A). The previous, informal definition of pre-XDR TB was MDR TB plus additional resistance to any fluoroquinolone, or any second-line injectable, but not both, whereas the definition of XDR TB from 2006 was TB resistant to any fluoroquinolone and to >1 of 3 second-line injectable drugs (capreomycin, kanamycin, and amikacin), in addition to MDR TB. MDR, multidrug-resistant; XDR, extensively drug-resistant.

Our study showed that XDR TB by the current definition is associated with exceptionally poor treatment outcomes, considerably worse than XDR TB by the former definition. The new XDR TB definition is applicable to fewer patients than the former definition, and treatment options are more limited. Veziris et al. (*8*) also observed a decrease in XDR TB patients using the revised definitions. Still, as use of bedaquiline and linezolid increases globally, resistance to those drugs will undoubtly increase, resulting in a higher number of XDR TB patients in the future. Better treatment outcomes for MDR TB have been associated with the use of bedaquiline, linezolid, and fluoroquinolones (*2*,*9*), and studies have shown worse outcomes for patients with bedaquiline resistance (*10*). Previous exposure to those drugs (i.e., bedaquiline, linezolid, fluoroquinolones) has been associated with worse patient outcomes compared with patients without previous exposure (*5*). Altogether, those factors explain the treatment success of only 31% for XDR TB, even lower than that recently found in a meta-analysis involving 10,223 XDR TB patients (94 studies, 26 countries) using the former definition, which together showed a pooled successful treatment outcome of 44% (95% CI 38%–50%) (*3*). That review found a XDR TB treatment success of 6% and 25% in 2 studies from Ukraine (n = 126).

The current definitions of pre-XDR TB and XDR TB, recommended since early 2021, are undoubtly more relevant than the former definitions, given they take into account WHO-recommended treatment regimens containing bedaquiline, pretomanid, linezolid, and moxifloxacin. Worryingly, but not surprisingly, the new definitions are associated with exceptionally poor outcomes for XDR TB, indicating loss of effective drugs. Upscaling of drug susceptibility testing, assessment of acquired drug resistance, and availability of diagnostic tools and drugs are crucial to avoid a future increase in patients with very limited treatment options. Treatment strategies should be assesed under programmatic conditions to improve understanding of the recommended treatment regimens, their implementation, and the effects on TB management globally.
